# Design of modified reference phase modulation based boost chopper fed fifteen level stepped DC link hybrid converter

**DOI:** 10.1038/s41598-024-52727-8

**Published:** 2024-02-01

**Authors:** R. Uthirasamy, S. Vishnu Kumar, Christo Ananth, Selvaraj David, Shruti Aggarwal, Syed Anas Ansar, Nitin Mittal, Lipika Gupta, Fikreselam Gared

**Affiliations:** 1 Department of Electrical and Electronics Engineering, Mahendra Engineering College, Namakkal, 637503 India; 2 Department of Electronics and Communication Engineering, Vel Tech Rangarajan Dr. Sagunthala R&D Institute of Science and Technology, Avadi, 600062 India; 3 Samarkand State University, 703004 Samarkand, Uzbekistan; 4 Chonnam National University, Gwangju, 61186 South Korea; 5 Department of Computer Science and Engineering, Thapar Institute of Engineering and Technology, Patiala, 147004 India; 6 Department of Computer Applications, Babu Banarasi Das University, Lucknow, 226028 India; 7 Skill Faculty of Engineering and Technology, Shri Vishwakarma Skill University, Palwal, 121102 India; 8 Chitkara University Institute of Engineering and Technology, Chitkara University, Rajpura, Punjab 140401 India; 9 Faculty of Electrical and Computer Engineering, Bahir Dar Institute of Technology, Bahir Dar University, Bahir Dar, Ethiopia

**Keywords:** Engineering, Electrical and electronic engineering, Energy infrastructure

## Abstract

A new fifteen-level stepped DC to AC hybrid converter is proposed for Solar Photovoltaic (SPV) applications. A boost chopper circuit is designed and interfaced with the fifteen-level hybrid converters specific to Electric Vehicles’ Brushless DC Motor (BLDC) drive systems. In chopper units, the output of solar panels is regulated and stepped up to obtain the nominal output voltage. In the stepped DC-link hybrid converter configuration, fifteen-level DC-link voltage is achieved by the series-operated DC-link modules with reduced electrical energy compression. From the comprehensive structure, it is anecdotal that the proposed topology has achieved minimum switching and power loss. Elimination of end passive components highlights the merits of the proposed hybrid systems. The reduction of controlled power semiconductor switches and gate-firing circuits has made the system more reliable than other hybrid converters. From the extensive analysis, the experimental setup has reported that 7% reduction in harmonics and a 54% reduction in controlled power switches than the existing fifteen-level converter topologies. Mitigation of power quality issues in the voltage profile of a fifteen-level multilevel hybrid converter is achieved through the implementation of dsPIC digital-controller-based gate triggering circuits.

## Introduction

Renewable energy resources are the key players in meeting real-time power demand. The recent downturn of global coal supply by 4% and the unexpected pandemic conditions have drastically changed the global economic conditions^1,2^. The power sector industry faced 40% of crashes due to the dependence on coal, resulting in rising fuel costs and making people move towards e-vehicles^3^. This pragmatic change in automobile usage has further burdened the need for electricity. It is understood that there is a recent surge in the practice of charging EVs using SPV set up^4–7^. Considering the limitation in the research perspectives^8^, an SPV-based charging setup is proposed for EVs. A lot of research activities have taken place to improve the efficiency of EVs; to bridge the gap between the development and utilization of the power semiconductor devices on EVs; researchers are developing a number of converter modules with chopper and inverter units^9–15^. As an alternative to three-level electric source converters, investigators use Multi-Level Inverters (MLIs) to utilize renewable resources^16–18^. It is imperative to study the merits of MLIs, that have facilitated the competent boundary with many electric utilities, such as (i) solar-powered industrial Power Supply Systems, (ii) operation of variable Frequency Drives (VFD), (iii) structuring of common DC bus, (iv) peak load and power loss compensator and (v) peak load demand management system^19–21^. Though MLIs have evidence of their remarkable merits from the researcher’s side, the practical applications are restricted due to high (i) controlled switching components, (ii) power supply and DC source units and (iii) gate driver circuits. To overcome the limitations of traditional MLIs, hybrid converters are implemented for high-power applications^22–24^. In conventional systems, to obtain the nominal output voltage in the Cascaded Multilevel Inverter (CMLI), a boost chopper is generally used between the DC sources and H-bridges. However, the efficiency and handling of CMLI and Boost Cascaded Multilevel Inverter (BCMLI) configurations get degraded because of a higher number of controlled power switches and DC sources. Thus, certain modifications in conventional CMLI and BCMLI systems are envisioned based on the 15-level hybrid converter SPV-based charging system^25–28^. The proposed system introduces two isolated DC-to-DC and back-end inverters for shaft load and auxiliary loads to reduce controlled power semiconductor switches and solar PV panels/sources. The nominal output voltage is obtained by introducing the boost chopper circuit in between the DC sources and DC link inverter -DC input voltage is boosted to the nominal voltage level by the boost chopper network. This reduces the number of DC sources and power switches, and using a three-level DC link chopper module provides the extended boost capability to the back-end inverter module.

The structure of the stepped DC link, DC to DC converter, is represented in “Introduction” section. The proposed hybrid inverter is presented in “Structure of 15-level boost hybrid converter system” section. The design of SPV with boost chopper for auxiliary load systems is addressed in Modes of operation section. Comparative analysis with traditional MLIs and hybrid converters is made in “Modified MCSPWM switching technique” section. The simulation and experimental results of three-level boosted 15-level hybrid converters are addressed in “Results and analysis” section. In conclusion, the observations and scope for further improvements are addressed.

**Figure 1:**
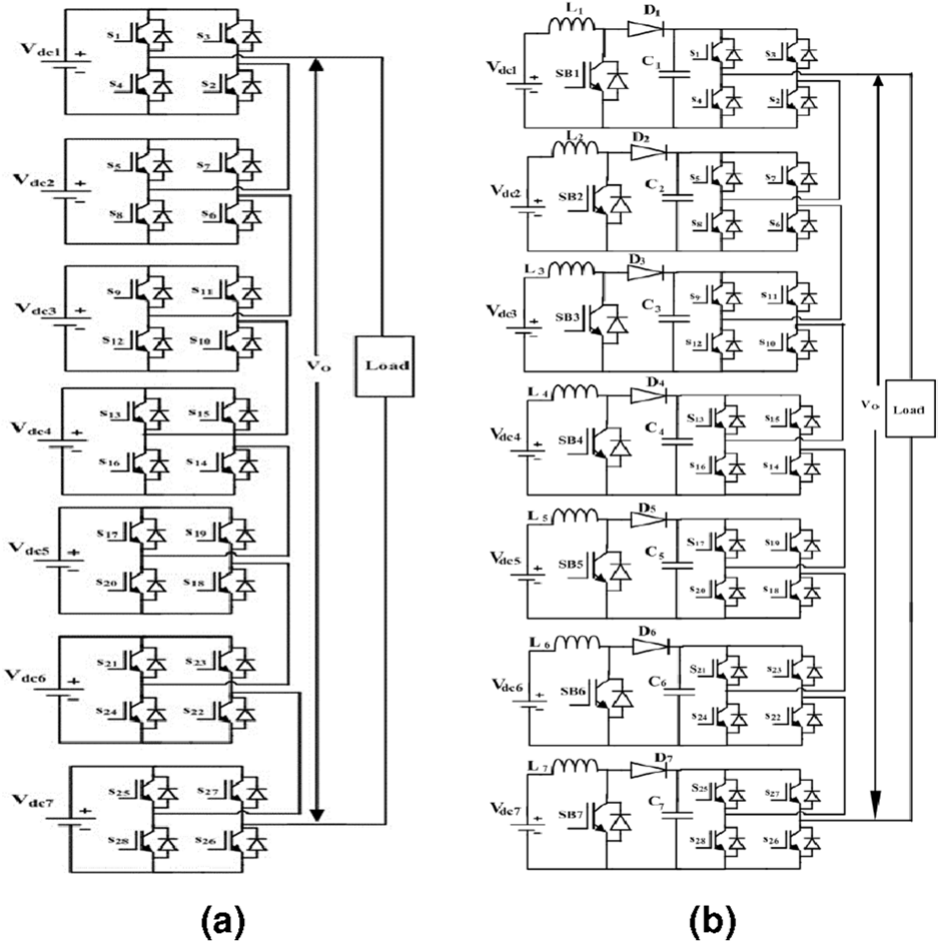
(**a**) Seven-layer cascaded multilevel inverter. (**b**) Seven-level boost cascaded multilevel.

The structure of cascaded multilevel inverter and boost cascaded multilevel inverter circuits are represented in Fig. 1a and b, respectively. With Fig. 1a and b as a reference, the required number of DC sources and controlled switches to develop a 15-Level AC output voltage in a Cascaded Multilevel Inverter is derived using the following Eqs. (1) and (2). The number of solar PV panels/sources and controlled switches required to develop a 15-Level AC output voltage in boost cascaded multilevel inverter derived using the Eqs. (3) and (4).

1 $$\begin{aligned} N_{switches}= & {} (s * 4) \end{aligned}$$

2 $$\begin{aligned} N_{level}= & {} (s * 2) + 1 \end{aligned}$$

3 $$\begin{aligned} N_{switches}= & {} (s * 4) + s \end{aligned}$$

4 $$\begin{aligned} N_{level}= & {} (s * 2) + 1 + s \end{aligned}$$

## Structure of 15-level boost hybrid converter system

**Figure 2:**
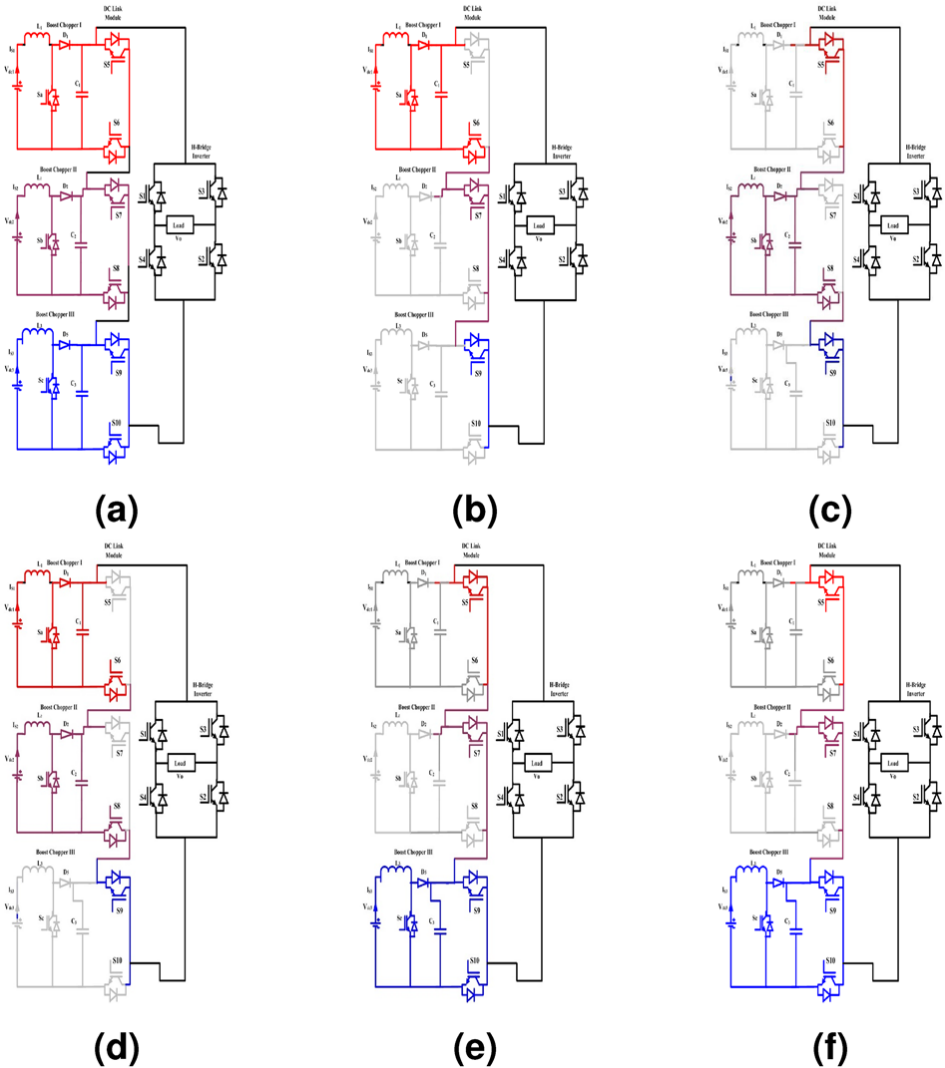
(**a**) Structure of 15-level hybrid converter. (**b**) Mode II and X operation. (**c**) Mode III and XI operation. (**d**) Mode IV and XII operation. (**e**) Mode V and XIII operation. (**f**) Mode VI and XIV operation.

The design structure of the 15-level boost hybrid converter is illustrated in Fig. 2a. The system is developed with PV panels as DC sources, a DC-to-DC boost chopper, a hybrid circuit and a two-level voltage source inverter. The number of PV panels and the semiconductor switches required to develop a 15-Level hybrid converter is estimated through Eq. (5).

5 $$\begin{aligned} N_{switches} = (s * 2) + s + 4 \end{aligned}$$

where, s is represents the input source

### Modes of operation

A 15-level boost hybrid inverter is operated for XVI modes to get hold of the desired output voltage. The transition manner of the hybrid converter is described in Table 1. Based on the switching table, the projected converter is operated at different modes as follows:

**Table 1:** Switching states.

Sub-multilevel switches						H-bridge switches				Output voltage Vo
$S_5$	$S_6$	$S_7$	$S_8$	$S_9$	$S_{10}$	$S_1$	$S_2$	$S_3$	$S_4$	
L	L	L	L	L	L	L	L	L	L	0
L	H	H	L	H	L	H	1	L	L	48
H	L	L	H	H	L	H	H	L	L	96
L	H	L	H	H	L	H	H	L	L	144
H	L	H	L	L	H	H	H	L	L	192
L	H	H	L	L	H	H	H	L	L	240
H	L	L	H	L	H	H	H	L	L	288
L	H	L	H	L	H	H	H	L	L	336
L	L	L	L	L	L	L	L	L	L	0
L	H	H	L	H	L	L	L	H	H	− 48
H	L	L	H	H	L	L	L	H	H	− 96
L	H	L	H	H	L	L	L	H	H	− 144
H	L	H	L	L	H	L	L	H	H	− 192
L	H	H	L	L	H	L	L	H	H	− 240
H	L	L	H	L	H	L	L	H	H	− 288
L	H	L	H	L	H	L	L	H	H	− 336

#### Mode I and mode IX operation

In mode I and IX operation, all power semiconductor voltage-controlled MOSFET switches are operated in OFF condition. Hence, the output voltage is estimated at zero level.

#### Mode II and mode X operation

In mode II and X operations, the input voltage of 36 V is boosted to 48 V through the activation of DC-to-DC converter switch $S_a$ with the desired duty ratio and the switches of proposed multilevel DC link converter $S_6, S_7, S_9$. The corresponding circuit for these operating modes is shown in Fig. 2b.

#### Mode III and mode XI operation

In mode III and XI operations, the input voltage of 72 V is boosted to 96 V through the activation of DC-to-DC converter switch $S_b$, and the switches of proposed multilevel DC link converter switches $S_5, S_8, S_9$. The equivalent circuit for these operating modes is shown in Fig. 2c.

#### Mode IV and mode XII operation

In mode IV and XII operations, the input 36 V is boosted to 48 V, 72 V is boosted to 96 V through the activation of DC-to-DC converter switch $S_a \; and \; S_b$, and the switches of proposed multilevel DC link converter switches $S_6, S_8, S_9$. The corresponding circuit for the operating modes is illustrated in Fig. 2d.

#### Mode V and mode XIII operation

In mode V and XIII operations, the input voltages of 36 V are boosted to 48 V, 72 V is boosted to 96 V through the activation of DC-to-DC converter switch $S_c$, and the switches of proposed multilevel DC link converter switches $S_5, S_7, S_{10}$. The equivalent circuit for these operating modes is shown in Fig. 2e.

At t = $T_{ON5}$, the DC-to-DC converter switch $S_c$ is activated. The boosted voltage across the inductor $L_3$ is expressed as in Eq. (6).

6 $$\begin{aligned} V_{dc3} = L_3 \frac{I_6 -I_7}{T_{ON5}} \end{aligned}$$

And the energy input to the inductor $L_1$ is expressed as Eq. (7).

7 $$\begin{aligned} E_{i3} = V_{dc3} \cdot I_{s3} \cdot T_{ON5} \end{aligned}$$

At t = $T_{OFF5}$, the DC-to-DC converter switch $S_c$ is de-activated, and the typical output voltage of the boost-chopper III circuit can be expressed using Eq. (8).

8 $$\begin{aligned} V_{ob3} = V_{dc3} + L_3 \frac{dI_{s3}}{T_{off3}} \end{aligned}$$

And the energy consumed by the load is given by Eq. (9)

9 $$\begin{aligned} E_{03} = (V_{ob3} - V_{dc3})I_{s3} \cdot T_{off3} \end{aligned}$$

#### Mode VI and mode XIV operation

In mode VI and XIV operations, the input voltage of 36 V is boosted to 48 V, and 144 V is boosted to 192 V through the activation of DC-to-DC converter switches $S_a$ and $S_c$, and the switches of proposed multilevel DC link converter switches $S_6, S_7, S_{10}$. The equivalent circuit for these operating modes is shown in Fig. 2f.

At t = $T_{ON6}$, the DC-to-DC converter switches $S_a$ and $S_c$ are activated. The energy input to the inductors is expressed using Eq. (10).

10 $$\begin{aligned} E_{i5} = (V_{dc1} + V_{dc3}) \cdot (I_{s1} + I_{s3}) \cdot T_{ON6} \end{aligned}$$

At $t= T_{OFF6}$, the DC-to-DC converter switches $S_a \; and\; S_c$ are deactivated, and the current through inductors reduces gradually. The energy consumed by the load system is calculated by Eq. (11).

11 $$\begin{aligned} E_{o5} = V_{ob1} + V_{ob3} - (V_{dc1} + V_{dc3}) (I_{s1}+I_{s3}) \cdot T_{off6} \end{aligned}$$

At $t= T_{ON7}$, energy input to the inductor $L_2$ is expressed and inductor L3 is expressed using Eq. (12).

12 $$\begin{aligned} E_{i7} = (V_{dc2} + V_{dc3}) \cdot (I_{s2} + I_{s3}) \cdot T_{ON7} \end{aligned}$$

At $t= T_{OFF7}$, the DC-to-DC converter switches $S_b \; and \; S_c$ are deactivated. At this instant, energy can consume by the load system is calculated by Eq. (13).

13 $$\begin{aligned} E_{o7} =(V_{ob2}+V_{ob3}) - (V_{dc2} + V_{dc3}) (I_{s2} + I_{s3}) \cdot T_{ON7} \end{aligned}$$

At $t= T_{ON8}$, the DC-to-DC converter switches $S_a, S_b \; and \; S_c$ are turned ON and the current through inductors raises linearly. At this instant, the energy inputs to the inductors can be expressed by Eq. (14).

14 $$\begin{aligned} E_{i8} =(V_{dc1} + V_{dc2} + V_{dc3}) \cdot (I_{s1} + I_{s2} + I_{s3}) \cdot T_{ON8} \end{aligned}$$

At $t= T_{OFF8}$, the DC-to-DC converter-controlled switches $S_a, S_b\; and \;S_c$ are turned OFF, and the current through inductors fall linearly. Thus, the energy transferred from the inductors to the DC-link module is given by Eq. (15).

15 $$\begin{aligned} E_{o8} = (V_{ob1} + V_{ob2} + V_{ob3}) - (V_{dc1} + V_{dc2} + V_{dc3})(I_{s1} + I_{s2} + I_{s3}) \cdot T_{off8} \end{aligned}$$

The output voltage of levels zero to seven is expressed as

16 $$\begin{aligned} V_{o1}= & {} V_{obc1} \end{aligned}$$

17 $$\begin{aligned} V_{o2}= & {} V_{obc2} \end{aligned}$$

18 $$\begin{aligned} V_{o3}= & {} V_{obc3} \end{aligned}$$

19 $$\begin{aligned} V_{o}= & {} V_{o1} + V_{o2} + V_{o3} = V_{obc1} + V_{obc2} + V_{obc3} \end{aligned}$$

The output voltage of levels zero to 15 is expressed as

20 $$\begin{aligned} V_{o1}= & {} - V_{obc1} \end{aligned}$$

21 $$\begin{aligned} V_{o2}= & {} - V_{obc2} \end{aligned}$$

22 $$\begin{aligned} V_{o3}= & {} - V_{obc3} \end{aligned}$$

23 $$\begin{aligned} V_{o}= & {} [V_{o1} + V_{o2} + V_{o3}] = -[V_{obc1} + V_{obc2} + V_{obc3}] \end{aligned}$$

Total Standing Voltage (TSV) of the proposed unit is calculated as

24 $$\begin{aligned} TSV = \biggl ( \frac{7s^2+5s-2}{2} \biggl )V \end{aligned}$$

From the Fig. 4c, it is inferred that the magnitude of 3rd, 5th, 7th and 9th order harmonics are 3 V, 2.8 V, 2 V and 2.7 V respectively. Hence the switching loss is 10.5 V, which is about 3%. Power Loss of the stepped DC link module is 20.5 V, thus the Total System Loss is 31 V. Load current of the system is 4.39 A for the rated current of 5 A. The average loss of the proposed system considered is 136 W. In the conventional systems, H-Bridge inverter switches are subjected to hard switching due to steady state DC link voltage across the switches in all time period. In the proposed system DC link voltage has zero crossing at every 10ms as shown in the Fig. 6b. The voltage stress across the inverter switches is reduced and hence the TSV is reduced.

## Modified MCSPWM switching technique

The entire system is operated optimistically through the modulation technique. The literature shows DC link systems can be triggered through phase modulation or level shift modulation^29,30^. In the proposed work, phase shift modulation techniques are adopted to operate the systems of DC link and inverter switches. Generally, carrier and modulating signals play a major role in pulse generation in the phase modulation schemes.

The vertical carrier distribution techniques are defined as level shifted (LS-PWM), which includes phase disposition (PD-PWM), phase opposition disposition (POD-PWM) and alternative phase opposition disposition (APOD-PWM). In the proposed system, reference signal is modified from the sine wave. Modified reference signals are used as reference signal hence in the modified signal, elimination of harmonics profile is maximum. Authors have done a exact modification in the peak amplitude of the sine wave reference signal.

**Figure 3:**
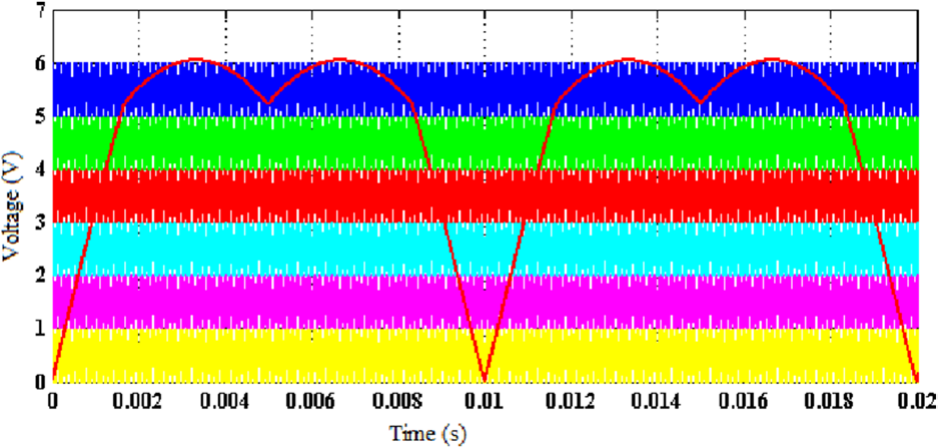
MRPOD PWM.

Modified reference signals are used as reference signals; hence, the elimination of the harmonics profile is maximum in the modified signal. As a researcher, the elimination or the reduction of hardware component play major design criteria. Optimizing techniques facilitate reducing the higher order harmonics to some extent of lower order harmonics. Selective harmonic elimination is achieved through the optimizing techniques adopted for the converter system. Figure 3 represents the Modified Reference Phase Opposing Disposition Pulse Width Modulation (MRPODPWM).

Mathematical expressions for the MRPOD-PWM switching scheme for DC link switching are formulated using space vectors.

25 $$\begin{aligned} V_a = V_m \sin \omega t \end{aligned}$$

Modulation index is expressed using equation

26 $$\begin{aligned} m_i = \frac{V_1}{V_2} \end{aligned}$$

The output voltage of DC link module is given by equation

27 $$\begin{aligned} V_{dc} = \frac{V_2}{2/\pi } * (m-1) \end{aligned}$$

where, $V_1$ is the peak value of fundamental voltage, $V_2$ is the peak value of fundamental voltage generated by the stepped DC link switches and Vdc is the DC link voltage provided by the asymmetrical source.

Modified reference signal is generated by calculating the sectors at each level. The procedure to calculate the sector points of the modified reference signal are given using equations

28 $$\begin{aligned} \text {For } m= & {} 1 \quad \quad V^*_a = T_1 + T_2 + T_0/2 \end{aligned}$$

29 $$\begin{aligned} \text {For } m= & {} 2 \quad \quad V^*_a = T_1 + T_0/2 \end{aligned}$$

30 $$\begin{aligned} \text {For } m= & {} 3 \quad \quad V^*_a = T_0/2 \end{aligned}$$

31 $$\begin{aligned} \text {For } m= & {} 4 \quad \quad V^*_a = T_0/2 \end{aligned}$$

32 $$\begin{aligned} \text {For } m= & {} 5 \quad \quad V^*_a = T_2 + T_0/2 \end{aligned}$$

33 $$\begin{aligned} \text {For } m= & {} 6 \quad \quad V^*_a = T_1 + T_2 + T_0/2 \end{aligned}$$

34 $$\begin{aligned} \text {For } m= & {} 7 \quad \quad V^*_a = 0.5V_a \end{aligned}$$

The output voltage and current harmonics of the proposed system are analyzed for different amplitude modulation indices ($M_a$ = 0.8, 0.95, 1.0 and 1.2) and frequency modulation indices ($M_f$ = 180, 200 and 220). The proposed system reduced the harmonic content for $M_a$= 1 and $M_f$ = 200. For developing the firing pulses of DC link switches, $N_{level} -1$ pattern of triangular signals are compared with sinusoidal reference signal.

When the magnitude of reference signal $$(V_{ref})$$ is greater than triangular signals $$(V_{tri1}, V_{tri2}$$ and $$V_{tri3})$$ the corresponding pulses $$p_1, p_2$$ and $$p_3$$ are generated.

## Results and analysis

To authenticate the operation of the proposed configuration, the complete system structure is pretended by means of the MATLAB-SIMULINK tool and the recital of the circuit was recorded.

**Figure 4:**
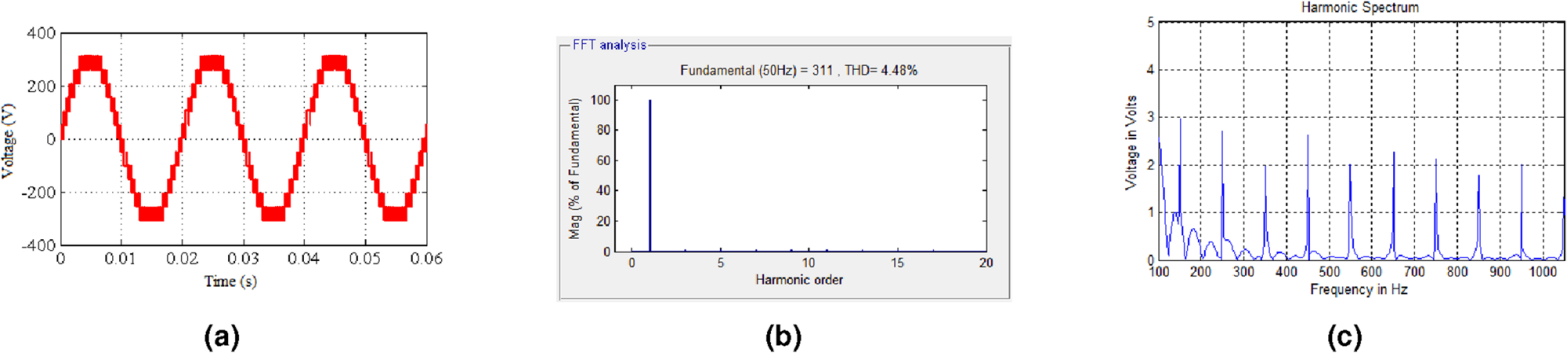
(**a**) Output voltage (level generation and H-bridge). (**b**) FFT-analysis of projected 15-level inverter. (**c**) Individual order voltage harmonic analysis (1-Phase).

The output voltage of the 15-level stepped DC to AC converter is shown in Fig. 4a. Each level of the output voltage is developed as per the logic behind the switching table. The time period of the output voltage is decided by the modulating signal utilized for the generation of PWM ON/OFF square waveforms. Harmonics analysis are made using the FFT algorithm^31,32^, and the THD profile of the output voltage is shown in Fig. 4b. From the analysis, it is inferred that the value of individual harmonics and THD are as per IEEE standard. Individual harmonics are analyzed and the order of the harmonics is represented in a graphical manner and the magnitude of individual harmonics is represented in Fig. 4c. From the analysis, it is inferred that the magnitude of $3rd, 5th, 7th \; and \; 9th$ order harmonics are 3 V, 2.8 V, 2 V and 2.7 V respectively.

The proposed stepped DC link-based inverter system is pretended, and the voltage stress (voltage amplitude ratio) across the inverter switches in terms of Power Spectral Density (PSD) is analyzed. PSD determines a signal’s power strength in the frequency province. Commonly, PSD is computed from the signal’s Fast Fourier Transform (FFT)^33^. Thus, the Power Density (PD) can be expressed as in Eq. (25).

35 $$\begin{aligned}PD \left(\frac{dB}{Hz}\right) = 20 log_{10}(X) \end{aligned}$$

**Figure 5:**
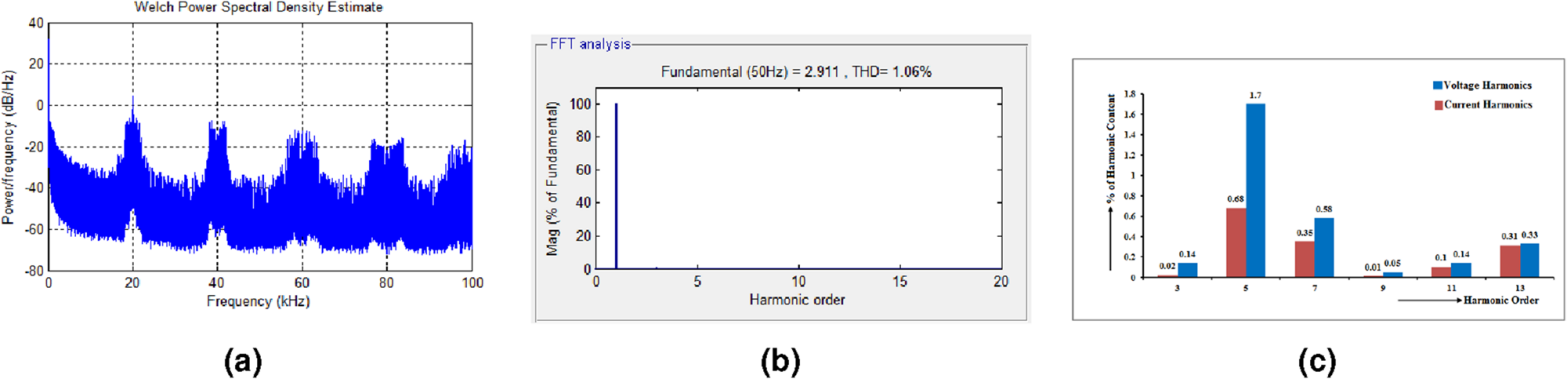
(**a**) Output voltage power density spectrum (single phase). (**b**) THD spectrum of load current. (**c**) Representation of voltage and current harmonics.

The proposed inverter selects the carrier wave with a 20 kHz frequency. Harmonic analysis is generated by switching occurrence multiples^34–36^. The Fig. 5a shows the PSD estimate for the proposed configuration at 20 kHz, 40 kHz, 60 kHz and 80 kHz as5 dB/Hz, − 8 dB/Hz, 16 dB/Hz, and 20 dB/Hz.

THD spectrum of the output load current of the proposed system is shown in Fig. 5b. Graphical representation of both voltage and current harmonics is shown in Fig. 5c. From the representation, it is inferred that the harmonic content present in the proposed converter system is low and it is concluded that the thermal effect on the load system due to inverter output supply is minimum and it increases the lifetime of the load system.

**Figure 6:**
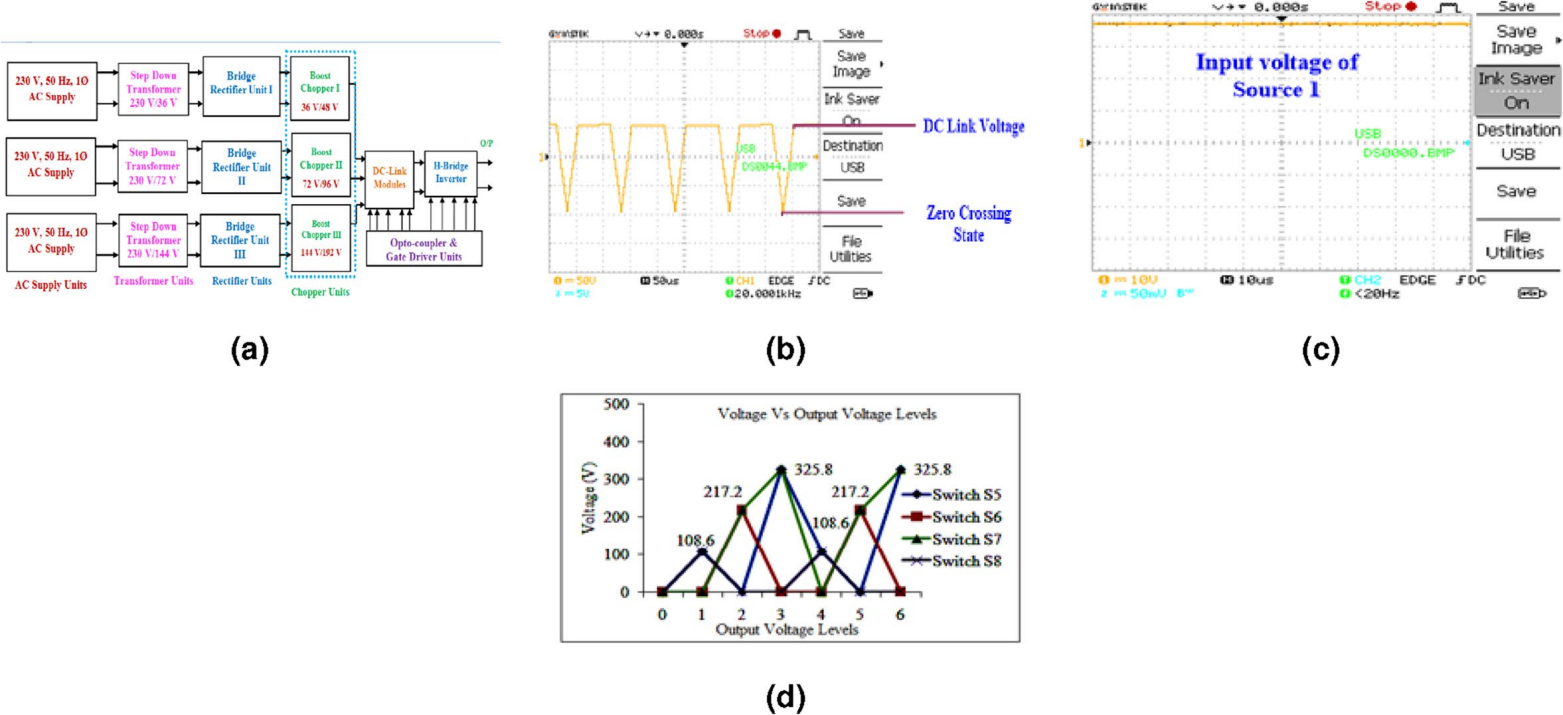
(**a**) Block diagram of the 15-level hybrid converter system. (**b**) DC link voltage. (**c**) Source voltage (Vdc1). (**d**) Blocking voltage of the DC link switches.

## Hardware system

The block diagram of the 15-level proposed hybrid system is shown in Fig. 6a. Figure 6b illustrates the DC Link voltage in the proposed system, the zero voltage magnitudes of unidirectional DC link voltage provide zero voltage switching for H-Bridge Inverter. From the Fig. 6c, it is inferred that the magnitude of $3_{rd}, 5_{th}, 7_{th}$ and $9_{th}$ order harmonics are 3 V, 2.8 V, 2 V and 2.7 V respectively. Hence the switching loss is 10.5 V, it is about 3%. Zero voltage switching states are represented in the Fig. 6b. In the conventional systems, H-Bridge inverter switches are subjected to hard switching due to steady state DC link voltage across the switches in all time period. In the proposed system DC link voltage has zero crossing at every 10 ms as shown in the Fig. 6b. Hence the voltage stress across the inverter switches is reduced. The DC link voltage of the proposed system is 150 V. DC Link switches are subjected to PWM modulation switching with a switching frequency of 2 kHz. Input Voltage to the Inverter system is 48 V, 96 V and 192 V $(V_{dcmax}= 336 V)$—in simulation model Input Voltage to the Inverter system is 36 V, 48 V and 60 V $(V_{dcmax}= 144 V)$—in hardware model. Boost ratio of the Proposed system is considered as 0.75 for all the boost chopper units. Figure 6c illustrates the input voltage. The proposed system is incorporated with step-down transformers, bridge rectifiers, DC-to-DC boost converters, a stepped DC Link hybrid module, a Two-level voltage source inverter, and gate driver circuits.

In the proposed system, the DC link module is connected for the following purposes:

1. To synchronize staircase waveform.
2. To achieve zero voltage/current switching, thus voltage tension across the switches is reduced.
3. To minimize the harmonic content
4. To interface the solar PV panels in series connection, thereby to achieve nominal output voltage through boost chopper.

The Fig. 6d pictorially represents the comparison results of MATLAB-Simulink simulation between input and output voltage. Suitable experimentation was conducted in MATLAB and the blocking voltage values have obtained across the power switches of the DC link module.

**Figure 7:**
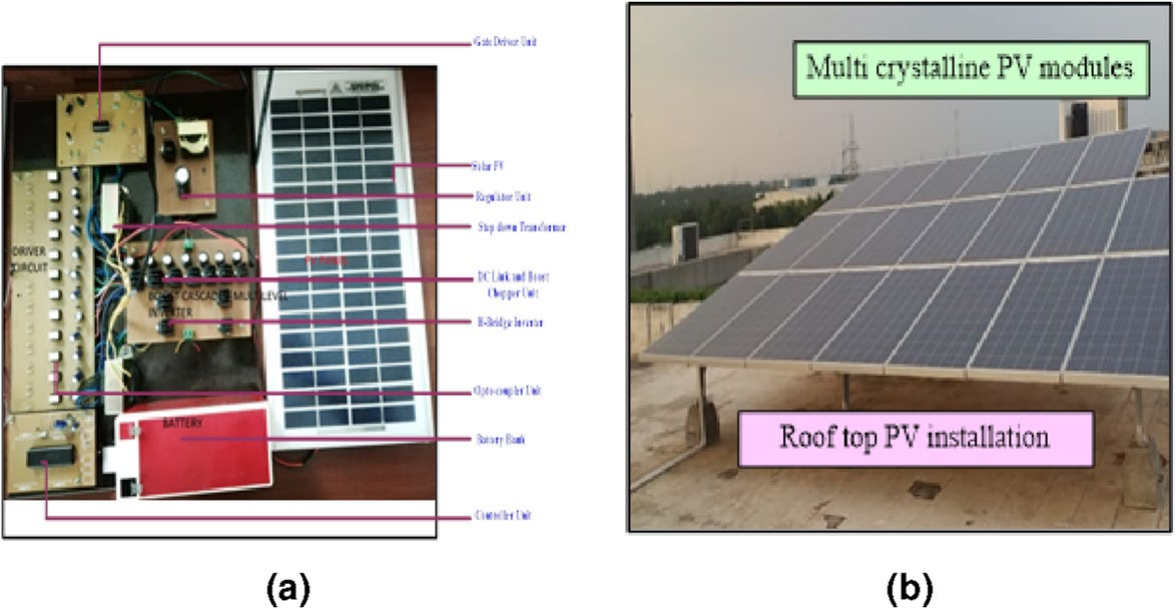
(**a**) Experimental setup. (**b**) SPV rooftop installation.

The performance of the projected converter is validated by fabricating the entire system as an experimental setup, as shown in Fig. 7a. The output power of the proposed converter is 750 W in experimental analysis and 1150 W in simulation analysis.

The 15-level stepped DC to AC hybrid converter configuration is designed and implemented for 150 V $(V_{max})$ output voltage. The prototype model consists of rectifier units, boost chopper units, controller units, driver units, DC link and H-Bridge inverter units. The rectifier units are designed using bridge rectifier MICBR1010 and a capacitive filter of 1000 μF. The outputs of rectifier units act as the input for the boost chopper units and battery banks.

The boost chopper units (Unit I and Unit II) are fabricated using IRF840 power MOSFET switches and passive components $(L_1=L_2$  = 2 mH and $C_1=C_2= 100 \; \upmu \text{F})$. The microcontroller (PIC16F877A) provides the control signals to the MOSFET driver circuit. The various features of microcontroller aid to achieve an effective control of the proposed system. The DC link module and H-bridge inverter systems are fabricated using IRF840 power MOSFET switches. Driver units consist of IR2110 Integrated Circuit (IC) and its biasing components and the isolation process is achieved by 6N135 IC. The technical specifications of IRF840 power MOSFET are entailed in Table 2. System specification is represented in Table 3. The proposed circuits are boost chopper unit, stepped DC link module, H-Bridge inverter module. 13 controlled switches are utilized for the proposed system; 3 controlled switches for Boost chopper unit, 6 controlled switches for stepped DC link module and 4 controlled H-Bridge inverter module. IR2110 IC is used as gate driver unit, PWM pulses are obtained from the port A and B of IR2110 IC. Three IR2110 ICs (One for boost chopper unit, one for stepped DC link module, one for H-Bridge inverter module are utilized for the proposed system. Each channel is isolated by optocoupler ICs. Totally 13 PWM signals are generated from the controller and the each signal is given to optocoupler IC. 13 PWM signals from the optocoupler ICs are given to three gate driver ICs. Load current of the system is 4.39 A for the rated current of 5A. The average loss of the proposed system considered as 136 W. Hence the Efficiency of the Proposed system is 91.2% (Table 4).

**Table 2:** Technical specifications of IRF840 MOSFET.

S. no.	Specifications		Range
1	Drain-to-source break down voltage	$V_{DS}$	500 V (min)
2	Static drain-to-source on-resistance	$R_{DS}$	0.85 $\Omega$
3	Source current	$I_S$	8.0 A
4	Gate-source voltage	$V_{GS}$	± 20 V
5	Power dissipation	$P_D$	125 W
6	Diode forward voltage	$V_{SD}$	2.0 V
7	Gate-to-source forward leakage current	$I_{GSS}$	100 nA
8	Gate-to-source reverse leakage current	$I_{GSS}$	− 100 nA
9	Drain-to-source leakage current	$I_{DSS}$	25 μA
10	Turn-on delay time	$T_{d(on)}$	14 ns

**Table 3:** System specification.

S. no.	Parameters	Range
1	Supply voltage, V	220
2	Drive current, A	4.6
3	Line frequency, $H_z$	50
4	Rated power, W	750

Each unit’s performance is discussed. The hardware setup is designed with the following units,

1. Source units—Solar PV as illustrated in Fig. 7b and battery bank.
2. DC-to-DC converter—Boost chopper is designed using MOSFET switches (IRF840)
3. Stepped DC link converter unit—Designed using MOSFET switches (IRF840)
4. Inverter unit—Designed using MOSFET switches (IRF840)
5. Power supply unit—Designed using step-down transformers (230 V/6V, 230 V/12 V) regulator ICs (7805 and 7812). In the Proposed system, prototype model consists of rectifier units, boost chopper units, controller units, driver units, DC link and H-Bridge inverter units. Controller, Optocoupler and gate driver units required constant power supply. Controller, Optocoupler ICs required 5 V power supply and the Gate driver ICs required 12 V power supply. 7805 Linear Regulator IC is utilized for Controller, Optocoupler units and 7812 Linear Regulator IC is utilized for Gate driver circuits. The authors have designed and implemented the prototype model with separate isolation for both the ICs.
6. Gate driver unit—Designed using gate driver ICs (IR2110). Driver units consist of IR2110 Integrated Circuit (IC) and its biasing components. Opto-coupler unit- Designed using optocoupler ICs- and the isolation process is achieved by 6N135 IC.
7. Opto-coupler unit—Designed using optocoupler ICs
8. Controller unit—Designed using controller ICs (dsPIC)

**Table 4:** Comparison of the proposed work with existing work.

Parameter	^37^	^38^	^39^	^40^	^41^	^42^	^15^	Projected converter
Number of required DC sources	3	4	3	3	3	3	2	3
Number of required power electronics switches	12	12	12	8	14	18	10	13
Number of MOSFET drivers	6	6	6	3	6	10	3	3
TSV	176	153	No data	No data	No data	162	148	136
Voltage levels	7	9	7	7	7	7	7	15
Requirement at inverter (series connected DC sources or transformer)	Yes	Yes	Yes	Yes	Yes	Yes	No	No
Efficiency	85.3	88.4	82.1	No data	No data	86.4	90.9	91.2

**Figure 8:**
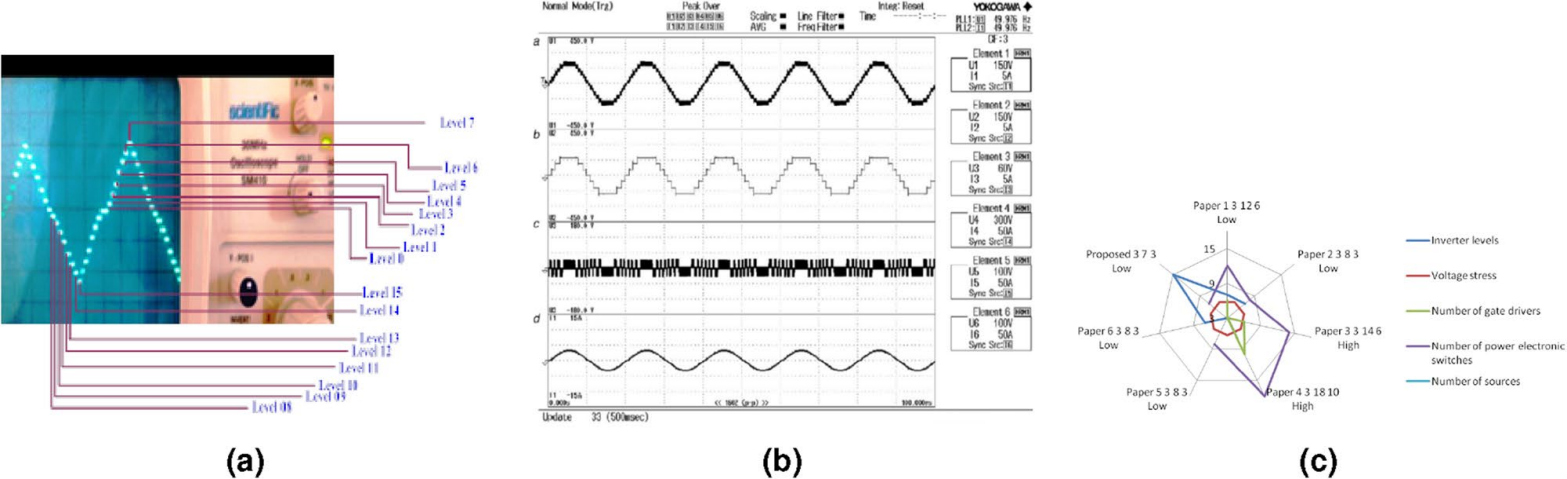
(**a**) Output voltage. (**b**) Output voltage and current waveform. (**c**) ScatterChart: qualitative analysis.

The experimental output voltage waveform of the proposed 15-Level hybrid inverter system is measured using CRO, as shown in Fig. 8a. From the output waveform, it is inferred that the output voltage is synchronized with 15-Level (One Zero, 7 Positive, and 7 Negative Levels).

The waveforms of experimental output voltage and current of the proposed 15-Level hybrid inverter system are also measured using power quality analyzer YOKOGAWA, as shown in Fig. 8b. From the analysis, it is observed that the magnitude and the frequency of the output voltage waveform are 325 Vmax and 49.976 Hz respectively. Element 1 in Fig. 8a represents the 15-level output voltage across the load. Element 2 in Fig. 8b represents the 7-level output voltage across the load (If two sources are connected). Element 3 in Fig. 8b represents the switching pattern for the DC link switches. Element 4 in Fig. 8b represents the load current.

The results of the comparison between the proposed work with other similar works in the literature are summarized in Table 2. It is inferred the proposed hybrid inverter gives better results, as illustrated in Fig. 8c, which also projects the qualitative analysis of the proposed CDDCLC work.

## Conclusion

Through this article, the authors communicate a single-phase 15-level DC-interface converter; to be used in solar-oriented Electric-Vehicle charging applications. The proposed analysis combines a DC-chopper circuit and inverter (H-connect) for efficient optimization of the controlled power semiconductor switches to accomplish the reduced harmonic profile. In comparison with H-bridge inverter systems, the proposed hybrid system works with condensed voltage stress; lessens number of switches and DC sources. The projected configuration requires only 3-DC sources for producing 15-level AC output. The proposed construction is suggested for economic power semiconductor switches used in SPV applications. Thus, MATLAB simulation and laboratory-prototype model prove the performance of the proposed ideology.

## Data Availability

The datasets used and analyzed during the current study are available from the corresponding author on reasonable request.
